# Exome-wide association study of treatment-resistant depression suggests novel treatment targets

**DOI:** 10.1038/s41598-023-38984-z

**Published:** 2023-08-01

**Authors:** Shrey B. Shah, Teja N. Peddada, Christopher Song, Maame Mensah, Heejong Sung, Mani Yavi, Peixiong Yuan, Carlos A. Zarate, Brian J. Mickey, Margit Burmeister, Nirmala Akula, Francis J. McMahon

**Affiliations:** 1 Human Genetics Branch, National Institute of Mental Health, National Institutes of Health, Bethesda, MD USA; 2 Rutgers New Jersey Medical School, Newark, NJ USA; 3 Stanford University School of Medicine, Stanford, CA USA; 4 Experimental Therapeutics and Pathophysiology Branch and Section on the Neurobiology and Treatment of Mood Disorders, National Institute of Mental Health, National Institutes of Health, Bethesda, MD USA; 5 Department of Psychiatry, Huntsman Mental Health Institute, University of Utah, Salt Lake City, UT USA; 6 Department of Psychiatry, University of Michigan, Ann Arbor, MI USA; 7 Michigan Neuroscience Institute and Department of Computational Medicine and Bioinformatics, University of Michigan, Ann Arbor, MI USA

**Keywords:** Genome informatics, Disease genetics

## Abstract

Treatment-resistant depression (TRD) is a severe form of major depressive disorder (MDD) with substantial public health impact and poor treatment outcome. Treatment outcome in MDD is significantly heritable, but genome-wide association studies have failed to identify replicable common marker alleles, suggesting a potential role for uncommon variants. Here we investigated the hypothesis that uncommon, putatively functional genetic variants are associated with TRD. Whole-exome sequencing data was obtained from 182 TRD cases and 2021 psychiatrically healthy controls. After quality control, the remaining 149 TRD cases and 1976 controls were analyzed with tests designed to detect excess burdens of uncommon variants. At the gene level, 5 genes, *ZNF248*, *PRKRA*, *PYHIN1*, *SLC7A8*, and *STK19* each carried exome-wide significant excess burdens of variants in TRD cases (q < 0.05). Analysis of 41 pre-selected gene sets suggested an excess of uncommon, functional variants among genes involved in lithium response. Among the genes identified in previous TRD studies, *ZDHHC3* was also significant in this sample after multiple test correction. *ZNF248* and *STK19* are involved in transcriptional regulation, *PHYIN1* and *PRKRA* are involved in immune response, *SLC7A8* is associated with thyroid hormone transporter activity, and *ZDHHC3* regulates synaptic clustering of GABA and glutamate receptors. These results implicate uncommon, functional alleles in TRD and suggest promising novel targets for future research.

## Introduction

Major depressive disorder (MDD) is a leading cause of disability worldwide, affecting over a quarter-billion people^1,2^. With often debilitating symptoms, MDD can pervade multiple aspects of mental, emotional, and physical health. Despite advances in drug and behavioral therapies, many patients suffer from treatment-resistant depression (TRD)^3–13^ that is nonresponsive to various available treatments. The prevalence of TRD ranges from 4 to 21%, depending on how it is defined; the most common definition is non-response to two or more antidepressant trials with adequate dose and duration of 4–6 weeks^14^.

Genetic studies of MDD aim to identify genes that point toward potential targets of pharmacological intervention and improve treatment outcomes. While genome-wide association studies (GWAS) have linked multiple common variants with MDD^15,16^, identifying genetic markers associated with antidepressant treatment outcomes has proven more difficult, possibly due to heterogeneity of the phenotype and limited sample sizes^17,18^. Although antidepressant response is estimated to be substantially influenced by genetic variation, with heritability up to 40%^19^, GWAS have found no replicable, genome-wide significant common variants associated with the phenotype^20–23^. This heritability gap suggests a role for uncommon genetic variants that are often missed by GWAS.

Here we investigated the hypothesis that uncommon, putatively functional genetic variants are associated with TRD. We performed a genetic association study that used exome sequencing to detect single nucleotide variants within the protein coding regions of genes, about 2% of the entire genome^24,25^. To enhance power, we focused on individuals with the most treatment-resistant forms of TRD and filtered for uncommon functional variants. We compared the overall burden of variants in cases to that in psychiatrically healthy controls, testing for differences primarily at the level of individual genes and pre-selected sets of genes representing key neurobiological and disease pathways. The results implicate uncommon genetic variants in TRD and suggest promising novel targets for future research.

## Materials and methods

### Study participants

A total of 182 unrelated individuals with TRD were selected for exome sequencing from 3 different sources: (1) 150 individuals diagnosed with MDD or bipolar disorder (BPD) whose depression symptoms failed to respond to > 2 antidepressants (failed lifetime antidepressant trials: median = 4; average = 5; range = 17) collected by the NIMH Experimental Therapeutics and Pathophysiology Branch (Protocol# NCT00024635 approved and monitored by the NIH institutional review board); (2) 24 individuals referred for electroconvulsive therapy at the University of Michigan (IRB protocol HUM00039846 approved and monitored by the University of Michigan institutional review board; a subset of participants in the Bluebird study^26^); and (3) 8 individuals selected from Level 4 of the Sequenced Treatment Alternatives to Relieve Depression study (STAR*D; Protocol# NCT00021528 approved and monitored by University of Texas Southwestern Medical Center at Dallas and the institutional review boards at each clinical site and regional center and the Data Coordinating Center and the Data Safety and Monitoring Board of the National Institute of Mental Health) on the basis of non-response to 3 or more consecutive treatments despite good physical health, absence of substance abuse, and reported good treatment adherence^27^. For controls, we used exome data from 2021 individuals with no psychiatric disorders collected by the Swedish Hospital Discharge Register^28^ and obtained from dbGaP (phs000473.v2.p2) in accordance with a Data Use Agreement monitored and approved by The Joint Addiction, Aging, and Mental Health Data Access Committee). All participants provided written informed consent and sample acquisition was performed in accordance with the guidelines stipulated by the above listed IRBs.

### Whole-exome sequencing data generation

DNA was extracted using Gentra Puregene Blood Kit (Qiagen N. V., Hilden, Germany). Libraries were captured using RocheNimbleGen (Roche Sequencing Solutions, Basel, Switzerland), SwiftBio (Integrated DNA Technologies, Inc., Coralville, IA, USA), or SureSelect (Agilent Technologies, Inc., Santa Clara, CA, USA). Libraries were captured from samples collected by the NIMH Experimental Therapeutics and Pathophysiology Branch and STAR*D using RocheNimbleGen (n = 158). Libraries were captured from samples collected by the University of Michigan via the Bluebird Study using SwiftBio (n = 24). Libraries were captured from samples collected by Swedish Hospital Discharge Register using SureSelect (n = 2021). Paired-end sequencing was performed on the Illumina GAII or HiSeq2000 platform (Illumina, Inc., San Diego, CA, USA) for both cases and controls.

### Mapping and variant calling

To minimize batch effects, sequence data from cases and controls were processed, mapped, and called jointly. Data quality (fastq files) was determined using Fastqc (v.0.11.9)^29^. Adapters were trimmed using FASTX-Toolkit (v0.0.13)^30^ and reads of mixed length (ranging from 63 to 100 bp) were mapped to the Ensembl human genome GRCh38.p13 using BWA-MEM^31^. PCR duplicates were removed using Picard MarkDuplicateSpark in the Genome Analysis Toolkit (GATK) (v.4.1.9.0)^32^. Base quality of reads was recalibrated using BaseRecalibrator and ApplyBQSR in GATK (4.1.9.0). HaplotypeCaller in GATK (4.1.9.0) pipeline was used to call the variants. After joint-calling, variants were filtered via GATK’s Variant Quality Score Recalibration. The data analysis pipeline is presented in Supplementary Fig. 1.

### Sample quality control (QC)

Samples with an atypical distribution of GC content, high (> 165 million) reads, and low (< Q20) Phred-scaled quality scores (n = 43) were excluded from further analyses. Variants with minor allele frequency (MAF) > 1% were used in PLINK (1.9) to verify sex (–check-sex), sample-level heterozygosity (–het), and relatedness (–genome)^33^. Samples with sex mismatch, relatedness ([Z0 < 0.4, Z1 > 0.2]), or high missingness (> 5%) were excluded (n = 7).

Twelve healthy control samples that were initially misclassified as TRD cases were corrected and included in the healthy control dataset for the downstream analysis, of which only seven remained after QC filtering. One sample from the healthy controls was excluded due to sample-specific artifacts. Samples outside 5 standard deviations from the centroid of the HapMapIII European cluster (n = 28) were excluded from the analysis (Supplementary Fig. 2). Details on samples removed during quality control are presented in Supplementary Table 1. Descriptive details of individuals remaining after quality control for cases and controls are presented in Table 1.

**Table 1:** Demographic and phenotype data for cases and controls after QC.

Reported race	Cases	Controls
White	98.0%	100.0%
Black/African American	0.7%	0.0%
Unknown	1.3%	0.0%
Reported sex		
Female	53.0%	49.0%
Male	47.0%	51.0%
Age at enrollment		
Average (Years)	45.5	Not available
SD (Years)	12.3	Not available
Diagnosis		
Major depressive disorder	84.6%	N/A
Bipolar I	9.4%	N/A
Bipolar II	6.0%	N/A

### Variant QC and annotation

A total of 1,339,135 variants were initially detected in this analysis. Genotypes with a genotype quality (GQ) < 20 and read depth (DP) < 10 were set to missing. Multi-allelic SNVs, SNVs < 10 bp from an indel, indels, intergenic SNVs, and variants with discordant genotypes between identical samples were excluded from the analysis. Variants not in Hardy–Weinberg Equilibrium (HWE *p* < 1E−6) in cases or controls were removed. Given that the cases and controls came from three different sources and where sequenced separately, locus-level heterozygosity was calculated for each set of individuals separated by source ((1) NIMH Experimental Therapeutics and Pathophysiology Branch and STAR*D; (2) Bluebird Study; (3) Swedish Hospital Discharge Register) and any variants with an observed heterozygosity of 1 were subjected to a permutation-based HWE test (HWperm). We verified the MAF of each SNV in our controls by comparing to the MAF of Non-Finnish European (NFE) in gnomAD genome (v2.1.1), gnomAD Exome (v2.1.1), and ExAC (v0.3) by a binomial test and 9550 variants with binomial test *p* < 0.05 were excluded (Supplementary Fig. 3). A locus missingness filter of 2% per batch and 5% for all cases and controls was applied. Known false-positive exome variants identified by Fajardo et al. were excluded from this study^34^. Details on variants removed during quality control are presented in Supplementary Table 2. Variant annotation was performed by ANNOVAR (v2020Jun08)^35^.

### Association analyses

We carried out association testing at three levels: single variants, genes, and sets of genes. False Discovery Rate correction was applied to resulting p-values using the Benjamini–Hochberg method.

### Variant level analysis and filtering

SKATBinary_Single function in SNP-set (Sequence) Kernel Association Test (SKAT; v2.0.1)^36^ package in R was used for single variant association analysis. SKAT_Null_Model_Moment Adjust with 100,000 resamplings was used to obtain the model parameters and residuals from the null model for small sample size. The efficient resampling method in the SKATBinary_Single function was used for calibrating *p* values of variants with low total minor allele counts^36^. The top 10 principal components of sample ancestry as calculated by PLINK (–pca) were included as covariates in the association model along with sex^33^.

#### Gene level analysis

Gene level analyses were conducted using three nested sets of variants: (1) Uncommon (control MAF < 5%), functional (annotated as non-synonymous and missense) variants (86,475 variants); (2) Uncommon (MAF < 5%), functional, damaging variants as annotated by ANNOVAR (v20210122)^35^ (59,549 variants); (3) Uncommon, functional, nonsense variants (annotated as “start loss”, “stop loss”, or "stop gain” in Ensembl) (2655 variants). For each set, the variants were grouped by gene, and processed with the SKATBinary function in SKAT. Utilizing the similar analysis as described above in variant level analysis, SKAT_Null_Model_MomentAdjust with a 100,000 resamplings was used as the null model. The efficient resampling method in SKAT was used to calculate empirical p-values of gene-level associations^36^.

#### Gene-set level analysis

Based on disease relevance and literature review, 41 gene sets were pre-defined and manually curated and selected for testing. Two of these gene sets were selected using DAVID, a functional annotation tool, using genes with *p* value < 0.05 as input^37^. Contents of tested gene-sets are presented in Supplementary Table 3. Uncommon functional variants from these selected gene-sets were input into Multi-marker Analysis of GenoMic Annotation (MAGMA; v1.10)^38^, a gene-set analysis tool to test for association, using the modifier multi = snp-wise to determine p values.

Overlaps between putative TRD-associated genes and those within PsychENCODE Cross Disorder Gene Coexpression modules and modules from Hartl et al. were tested utilizing a hypergeometric test^39,40^.

### Replication of top hits

Gene-level results were compared with the published top hits reported by three previous TRD GWAS^41–43^. Since full summary statistics were not available for those studies, the Sidak method was used for multiple test correction.

### Validation—Sanger sequencing

Uncommon variants in ZNF248, the only gene to be significant after Bonferroni correction, were validated in TRD cases using Sanger sequencing. Primers were designed using NCBI Primer-Blast (https://www.ncbi.nlm.nih.gov/tools/primer-blast/). PCR was performed using Taq PCR Core Kit (Qiagen N. V., Hilden, Germany). For size verification, an amplified PCR product was run on a 2% Agarose gel on E-Gel Power Snap Electrophoresis System (Thermo Fisher Scientific, Waltham, MA, USA). The PCR product was purified using the QIAquick PCR purification kit (Qiagen N. V., Hilden, Germany). Sanger sequencing was performed by Psomagen (Rockville, MD, USA).

## Results

After QC, exome sequencing data of 114 billion reads (~ 54 million reads per sample) in a total of 149 TRD cases and 1976 controls were included in this analysis. The Ti/Tv ratio and GC percentage were 2.75 and 49%, respectively. After filtering, a total of 189,497 variants were included.

### Variant level

There was no evidence of bias in the analysis, with an exome-wide lambda value of 0.9619 (Supplementary Fig. 4). No exome-wide significant variants were identified. This was expected in this sample of unrelated people given the heterogeneous nature of TRD. The Manhattan plot is shown in Supplementary Fig. 5. Nominally significant variants are presented in Supplementary Table 4.

### Gene level

**Figure 1:**
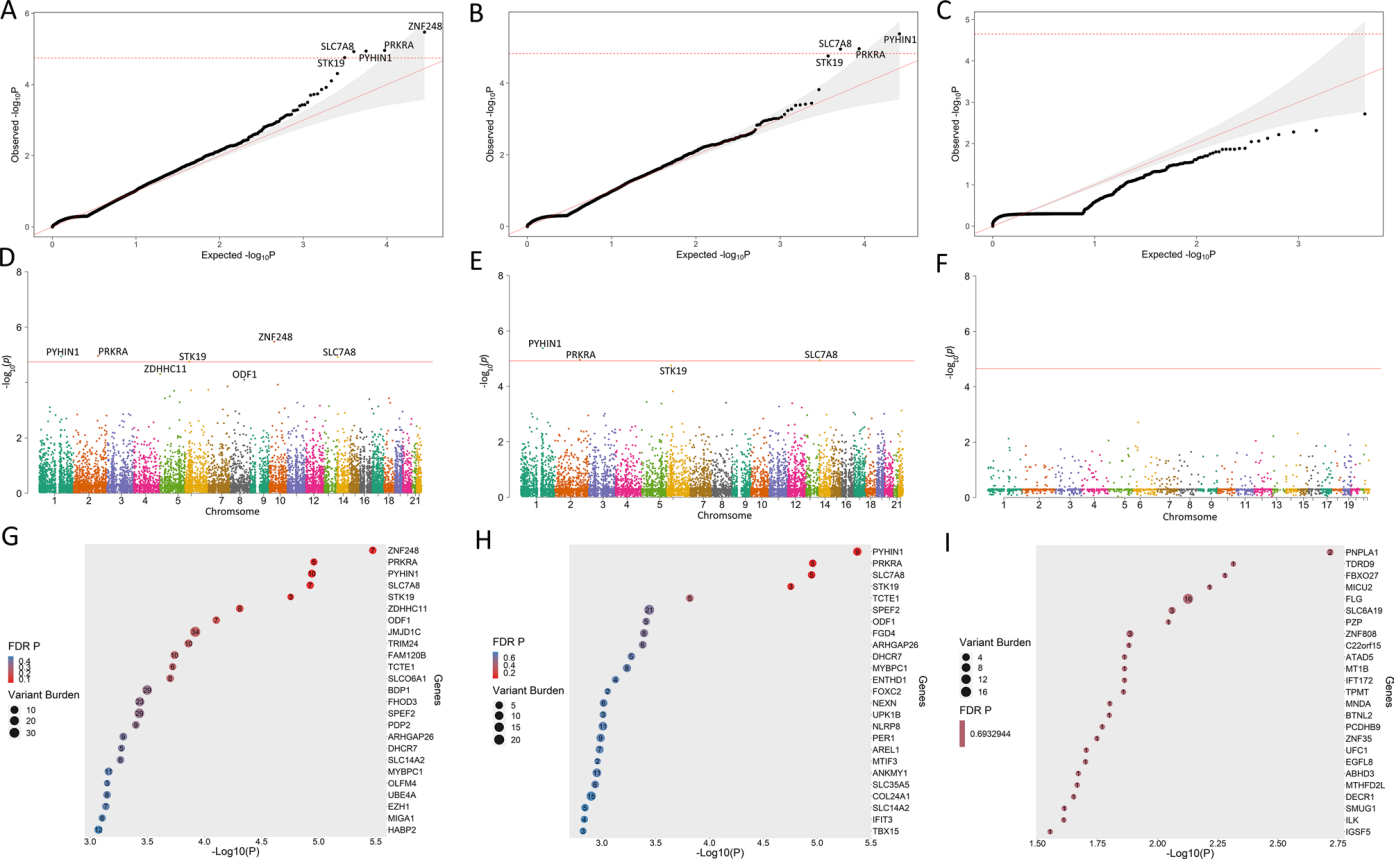
Gene level results. The red lines denote FDR 5% significance threshold. Top: Quantile–Quantile Plots of Uncommon (**A**) Functional, (**B**) Damaging, and (**C**) Nonsense Variants. Middle: Manhattan Plots of uncommon (**D**) Functional, (**E**) Damaging, and (**F**) Nonsense Variants. Bottom: Bubble Plots of uncommon (**G**) Functional, (**H**) Damaging, and (**I**) Nonsense Variants. The number inside of each circle denotes the number of variants scored in each gene.

Uncommon, functional variants in 14,083 genes were tested for association with TRD. There was no evidence of bias in the analysis (lambda = 0.9333; Fig. 1A). The gene-level Manhattan plot is shown in Fig. 1D. Five genes (*ZNF248, PRKRA, PYHIN1, SLC7A8, and STK19)* carried excesses of uncommon, functional variants in TRD cases at an FDR < 5%. These genes represent a variety of biological themes, consistent with the likely heterogeneous architecture of TRD (Table 2). Further filtering for damaging variants included few variants in each gene and did not yield any additional significant results (Fig. 1B–C, E–F, H–I). The top 50 genes associated with TRD are shown in a bubble plot in Fig. 1G and all nominally significant genes are shown in Supplementary Table 5.

**Table 2:** Gene-level association results.

Symbol	Gene name	Variants	Total carriers		*P* value	FDR Q	Depth of coverage	
			Cases (%)	Controls (%)			Cases	Controls
*ZNF248*	Zinc Finger Protein 248	7	18 (12.1)	52 (2.6)	3.4E−06	0.0418	52	37
*PRKRA*	Protein Activator of Interferon-Induced Protein Kinase	5	27 (18.1)	137 (6.9)	1.1E−05	0.0418	121	176
*PYHIN1*	Pyrin and HIN Domain Family Member 1	10	15 (10.1)	67 (3.4)	1.1E−05	0.0418	43	40
*SLC7A8*	Solute Carrier Family 7 Member 8	7	9 (6)	40 (2.0)	1.2E−05	0.0418	43	46
*STK19*	Serine/Threonine-Protein Kinase 19	3	9 (6)	12 (0.6)	1.8E−05	0.0495	45	35

### Gene set level

**Figure 2:**
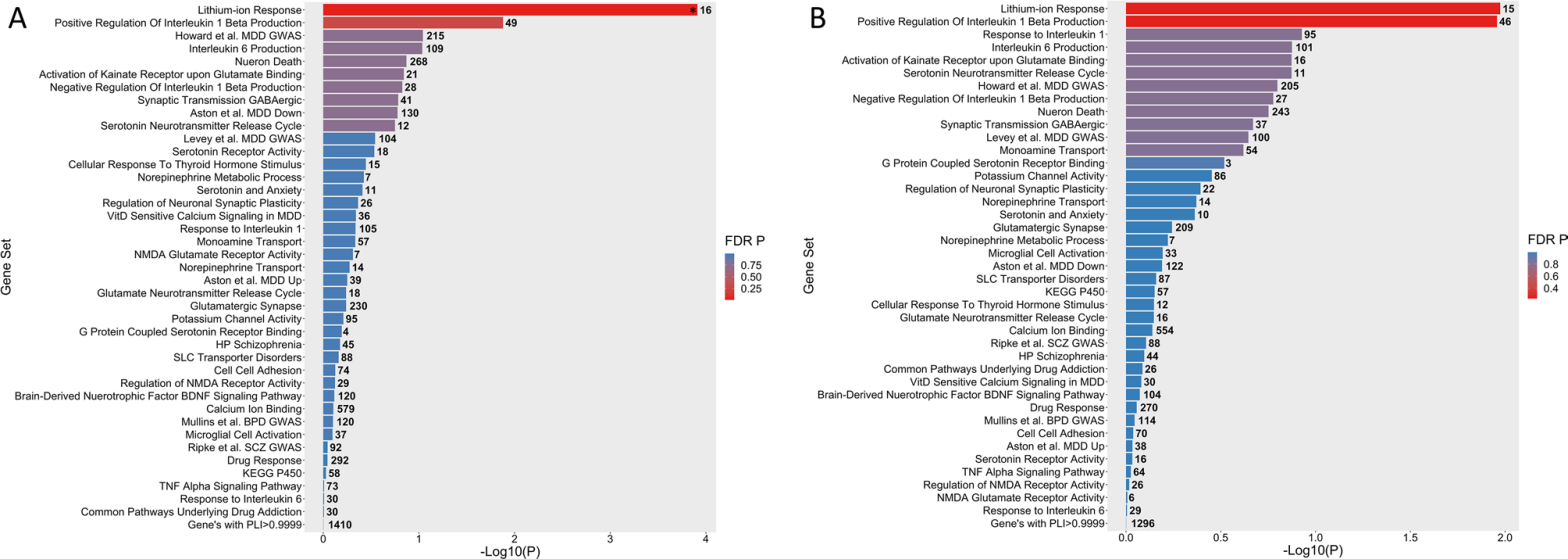
Gene set level results. The number adjacent to each bar denotes the number of genes contained within that gene set. (**A**) Bar Chart of Gene Set Analysis based on (**A**). Uncommon Functional and (**B**) Uncommon Damaging Variants. * denotes FDR q < 0.05.

Of the 41 gene sets tested (Fig. 2A), only one carried a significant excess burden of uncommon functional variants in cases after FDR correction. This gene set, GO:0010226: Response to Lithium Ions, comprised 75 variants in 16 genes and was associated with TRD at q = 5.038E−03. One additional gene set, GO:0032731: Positive Regulation Of Interleukin 1 Beta Production, was nominally significant before FDR correction. Additional filtering for damaging variants included too few variants in each gene-set and did not yield any additional significant results (Fig. 2B). Details on the gene sets tested are shown in Supplementary Table 6.

We also investigated 30 Cross Disorder Gene Coexpression Modules previously reported by PsychENCODE and 11 Coexpression network modules from Hartl et al. for association with TRD using the hypergeometric test^39,40^. None were significantly associated with TRD after FDR correction (Supplementary Table 7).

### Replication

Among the 54 unique genes associated with TRD reported in previous GWAS studies^41–43^, Zinc Finger DHHC-Type Palmitoyltransferase 3 (*ZDHHC3*) was supported in the present study at a Sidak-corrected *p* value < 0.05 (Supplementary Table 8).

None of the 5 significant genes were replicated in the only other large published exome-wide association study of antidepressant response^43^ (minimum *p* = 0.1339 for damaging variants in TRD vs response).

### Validation

The 12 cases with a contributing variant (rs11011379) in the gene *ZNF248* were selected for Sanger sequencing. All 12 putative heterozygotes were validated for this variant.

## Discussion

We conducted an exome-wide association study at the single variant, gene, and gene-set levels among individuals diagnosed with TRD and unaffected controls. The goal was to identify potentially causal variants and increase our understanding of the neurobiology of TRD.

The results suggest a role for uncommon, functional alleles in TRD. Five genes were found to carry significant excesses of uncommon, functional variants in TRD cases. Of the 22 genes in the lithium-ion response gene set, 16 genes also carried an excess of uncommon functional variants in TRD. Among the 54 genes most strongly associated with TRD in previous studies, *ZDHHC3* was also significantly associated with TRD in the present study.

The five genes that carried excesses of uncommon, functional variants in TRD cases represent a variety of biological themes, consistent with the likely heterogeneous architecture of TRD.

*ZNF248* is involved in DNA recognition, transcriptional activation, regulation of protein folding and RNA packaging, and lipid binding. A previous TRD GWAS reported association with a common SNP, rs2505705, that is an eQTL for ZNF248 and other genes, suggesting that common variants in this gene may also contribute to TRD^41^. Although the function of zinc finger proteins, including *ZNF248*, in MDD remains to be fully determined^44^, *ZNF248* is highly expressed in the brain. Another zinc-finger gene implicated by this study, *ZDHHC3,* regulates synaptic clustering of GABA and glutamate receptors. *STK19* resides in the MHC locus on chr 6p, a significant GWAS locus for schizophrenia, depression, and bipolar disorder^45^. *STK19* is one of the 4 genes in the RCCX cassette, previously implicated in schizophrenia^46^ and congenital adrenal hyperplasia, which itself is associated with increased risk for variety of psychiatric disorders^47^. *PYHIN1* (pyrin and HIN domain family member 1) is related to interferon regulation, involved in transcriptional regulation of genes important for cell cycle control, and has been linked to depressive behaviors induced by lipopolysaccharide in mouse models^48^. The *PRKRA* gene plays an important role in the inhibition of translation and induction of apoptosis pathways, and is implicated in neurodegenerative diseases related to inflammation^49–52^. *SLC7A8* encodes the Large neutral Amino acids Transporter small subunit 2 (LAT2) which enables thyroid hormone transporter activity. Deficient thyroid function can cause depressed mood and is generally treatable with adequate thyroid replacement. The use of triiodothyronine (T3) as a potential augmentation strategy in cases of TRD even without thyroid dysfunction, has been highlighted in several studies^53,54^.

Our gene-set analyses found that the lithium-ion response gene set (GO:0010226) was significantly associated with TRD. Lithium is a first-line treatment option in bipolar disorder, in either manic or depressed states, and often used as an augmentation treatment for antidepressants. Thus, a significant percentage of the cases in our study have used lithium. We unfortunately do not have data that indicates whether these cases are responders or non-responders. Furthermore, we only have these data for cases from the NIMH Experimental Therapeutics and Pathophysiology Branch and not for all our cases. With these limitations, our results suggest that lithium-response may explain why some individuals with MDD or BPD become treatment resistant, or that lithium neurobiology may be involved in treatment response in major mood disorders.

These results also highlight a role for immune response in TRD. Among the significant associations we detected, two genes (*PYHIN1* and *PRKRA*) were involved in immune response. Interestingly, one gene set (“Up Regulation of Interleukin 1 Beta”) was associated with TRD in the present study at nominal significance levels; however, due to the number of gene sets tested this association is very limited. These results are consistent with the growing body of evidence that neuroinflammation plays a role in psychiatric disorders^55–59^.

There have only been two previous exome wide association studies of TRD^43,60^. In the first study, Tammiste et al. in 2013 conducted a small (n = 10) exome wide association study of treatment non-response in MDD and found variants in bone morphogenetic protein 5 (*BMP5*) to be associated with non-response to a single treatment trial of escitalopram^60^. In the second study, Fabbri et al. in 2020 conducted a larger (n = 1209) exome wide association study comparing typical antidepressant response with TRD and found no exome-wide significant variants, genes, or gene sets^43^. The significant results in the present study, with a smaller case sample than Fabbri et al., may be attributable to power gains through a larger healthy control group compared to their smaller control group that also had MDD (responsive to antidepressants). Furthermore, the selection of severe TRD cases may enhance enrichment of uncommon, high-risk variants^61^. We selected severe samples from the fourth level of STAR*D, samples treated with electroconvulsive therapy (ECT), and samples with multiple failed treatment trials, in contrast to the more typical TRD cases studied by Fabbri et al.

This study has several important limitations. We focused on a relatively rare form of MDD, resulting in a relatively small sample of cases. Larger samples are needed, but the discovery of genes and gene sets in this study suggests that the “extreme phenotype” strategy we employed can be an effective means to identify uncommon genetic variants—even in modestly sized samples^62–64^. The varying definition of TRD across the case samples used in this study leaves some uncertainty as to which aspects of antidepressant treatment resistance were most influenced by uncommon variants. This heterogeneity is a necessary consequence of aggregating samples to increase sample size and power. The controls we used were psychiatrically healthy and did not include cases of typical antidepressant treatment responders. Thus, we cannot rule out that some of our findings reflect genetic risk for MDD itself, rather than TRD, but this is unlikely to account for most of our findings. We found no evidence that genes previously associated with MDD were over-represented among the TRD-associated genes we identified. Furthermore, the forms of TRD represented by the cases we studied are likely to be very uncommon in the general population and in the non-psychiatric controls we employed. Although the samples we studied used different capture libraries for cases and controls, we found no evidence of bias in the form of inflated p-values at the variant, gene, or gene-set level, with exome-wide lambda values close to 1. The cases and controls we used were of European ancestry; studies in more diverse populations are needed and may implicate different genes and mechanisms^65^.

In summary, by focusing on individuals with severe TRD and filtering for uncommon variants, we identified genes and gene-sets likely to predispose to this important psychiatric illness. The genes and gene-sets identified in this study suggest that transcriptional regulation, lithium response, thyroid hormone transporter activity, and immune response are all involved with TRD. These results highlight potential biological markers and mechanisms that could lead to new therapeutic targets in TRD. We hope that our findings will provide novel hypotheses for future investigations into the etiology of TRD. With the continual decrease in sequencing costs and a growing effort to build large biobanks and cohorts of patients with TRD^66^, exome sequencing analysis will be an increasingly important tool to better understand the complex trait of antidepressant treatment outcome.

## Supplementary Information

Supplementary Information 1.

Supplementary Information 2.

Supplementary Information 3.

Supplementary Information 4.

Supplementary Information 5.

Supplementary Information 6.

Supplementary Information 7.

Supplementary Information 8.

Supplementary Information 9.

Supplementary Information 10.

## Data Availability

The datasets generated and/or analyzed for the current study’s controls are available in the dbGaP repository, [phs000473.v2.p2]. The datasets generated and/or analyzed for this study’s cases are available in dbGaP, [phs003329.v1.p1].
