# Supplementary figures and images for: Exome-wide association study of treatment-resistant depression suggests novel treatment targets
Source: Sci Rep. 2023 Aug 1;13:12467. doi: 10.1038/s41598-023-38984-z (PMC10394052; doi:10.1038/s41598-023-38984-z)

Supplementary Figure 1

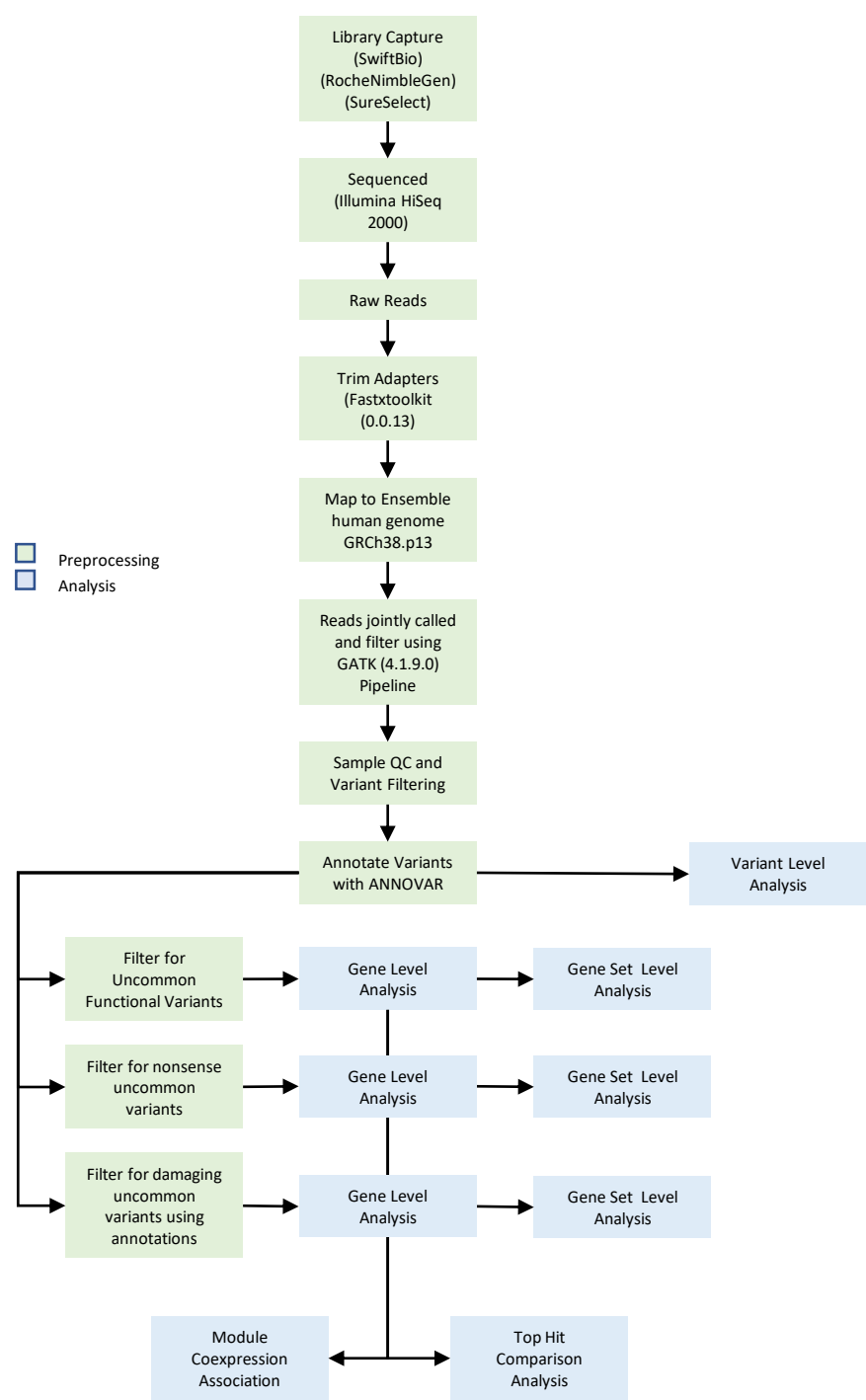

Supplementary Figure 2

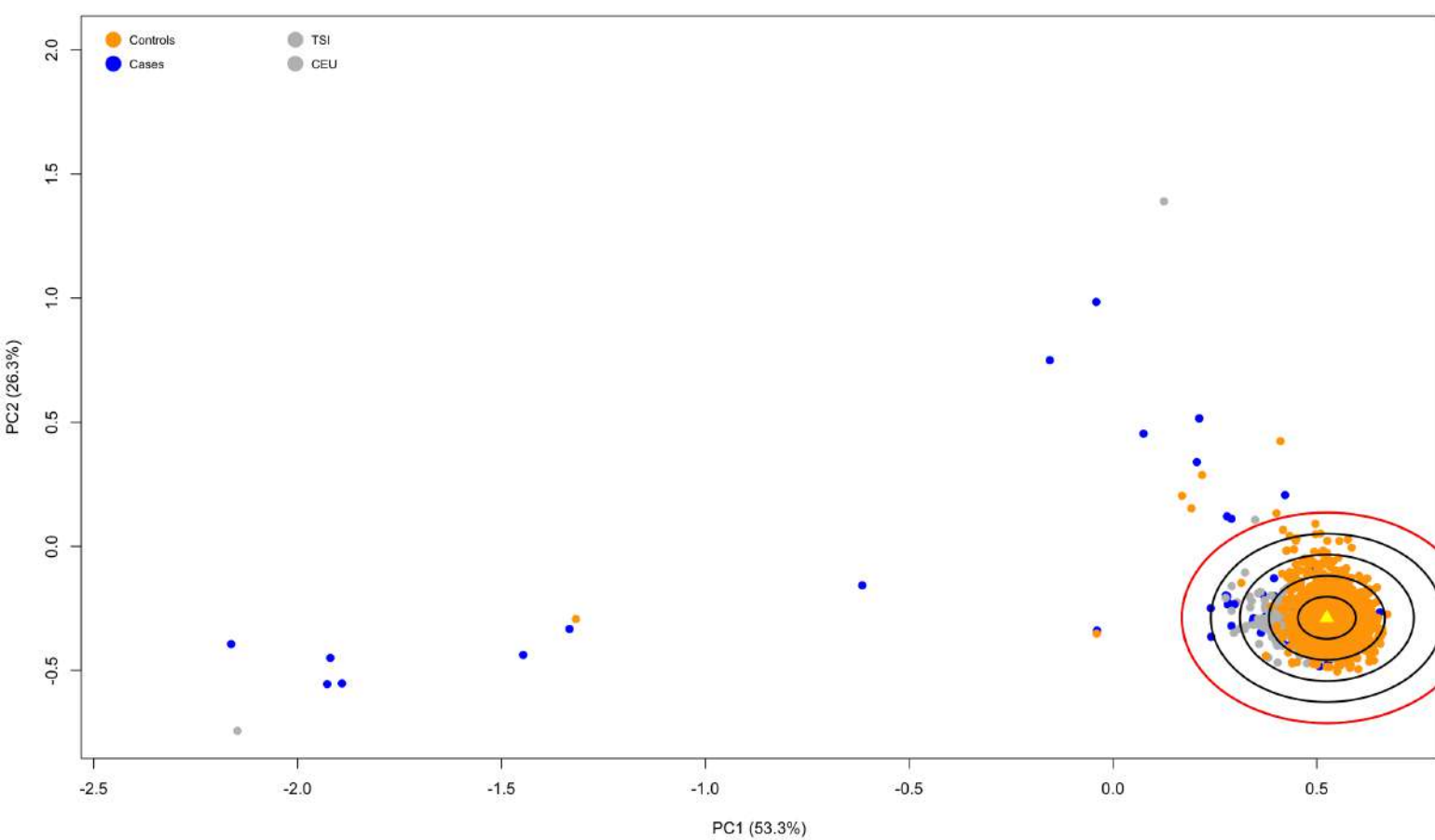

Supplementary Figure 3

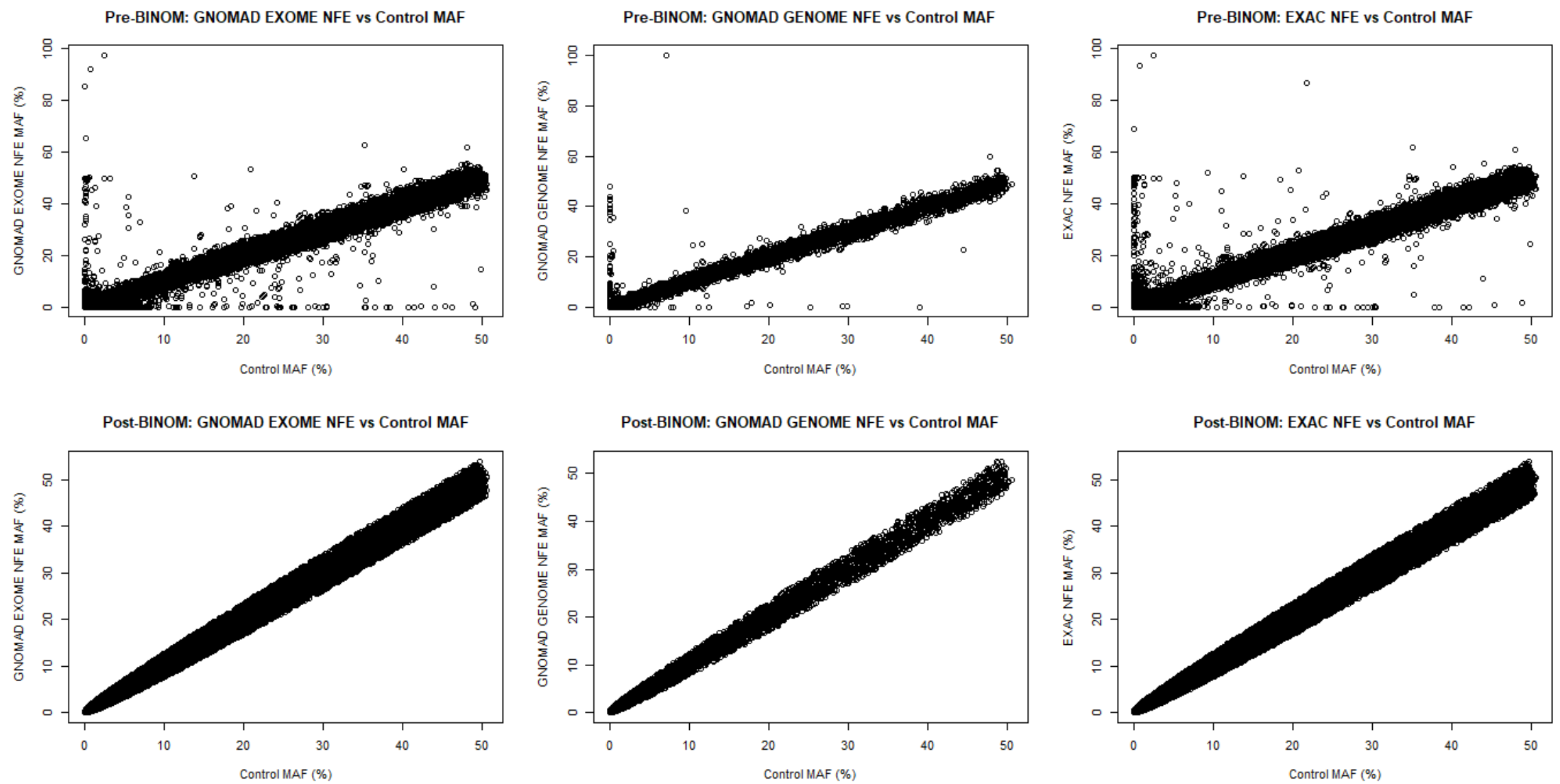

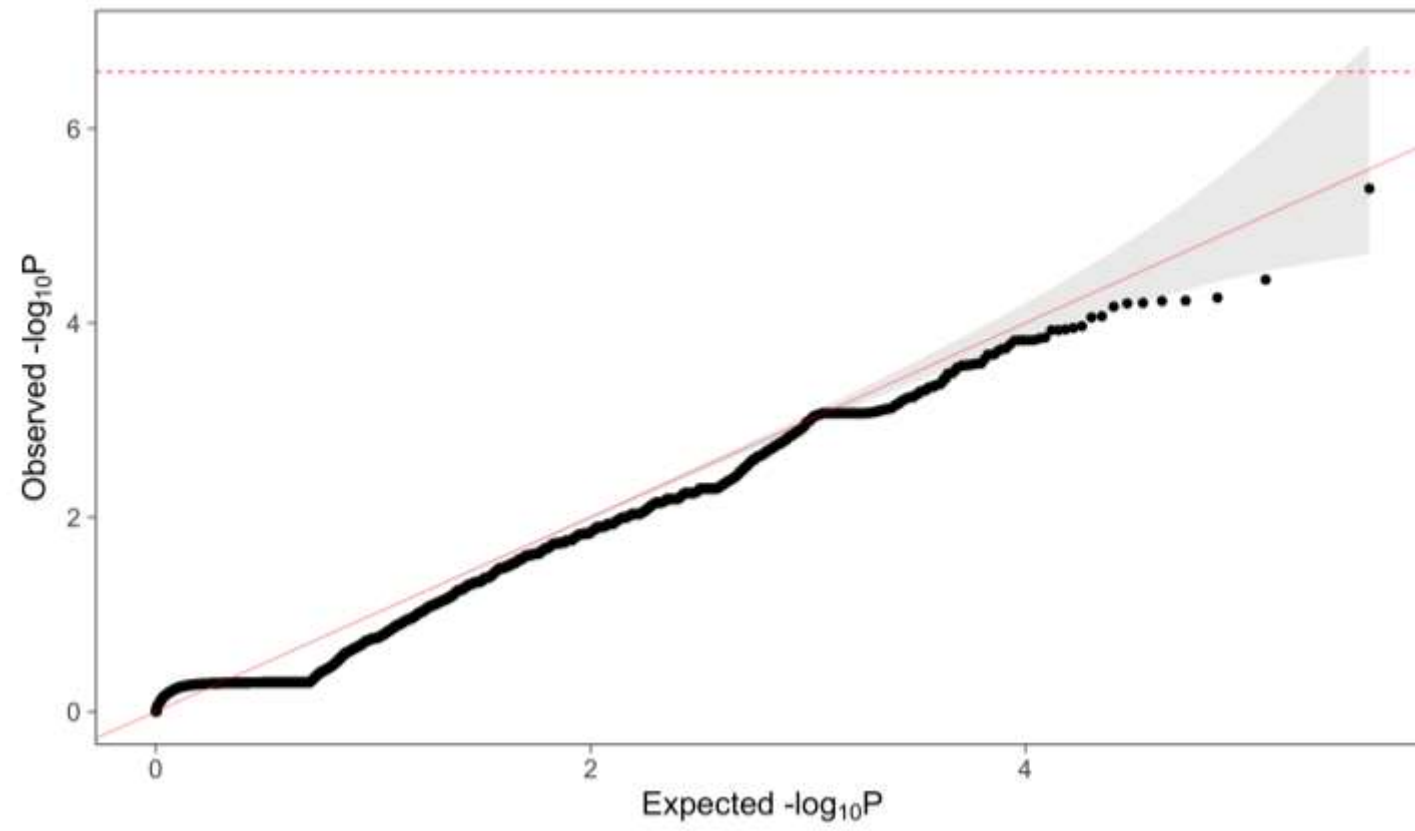

Supplementary Figure 5

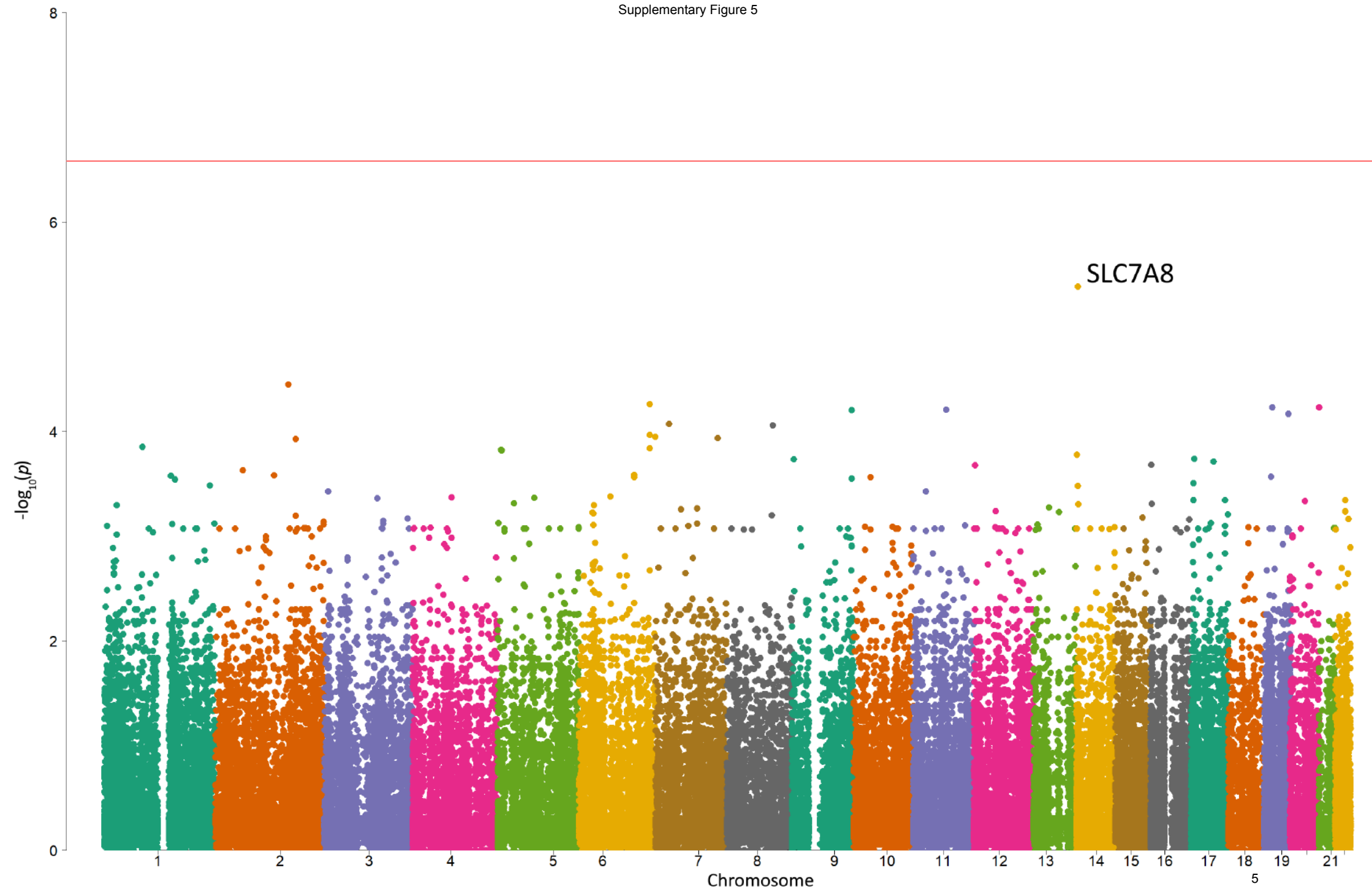

Supplement: Supplementary file 1 — Supplementary Information 1. [file 41598_2023_38984_MOESM1_ESM.pdf]
