# Supplementary material for: Exome-wide association study of treatment-resistant depression suggests novel treatment targets
Source: Sci Rep. 2023 Aug 1;13:12467. doi: 10.1038/s41598-023-38984-z (PMC10394052; doi:10.1038/s41598-023-38984-z)
Supplement: Supplementary file 2 — Supplementary Information 2. [file 41598_2023_38984_MOESM2_ESM.docx]

# Supplementary Information

Supplementary Figure 1. Analysis Pipeline.

Supplementary Figure 2. Ancestry PCA Plot with HapMapIII Data.

Supplementary Figure 3. Impact of gnomAD MAF filtering on MAF values in control samples

Supplementary Figure 4. SNV Level Quantile-Quantile Plot.

Supplementary Figure 5. SNV Level Manhattan Plot. Red line denotes the FDR 5% significance threshold.

Supplementary Table 1. Sample Quality Control Steps with numbers of samples excluded at each QC step.

Supplementary Table 2. Variant Quality Control.

Supplementary Table 3. Contents of Gene Sets Tested. Both the GO Term and the individual constituent genes within the gene set are listed.

Supplementary Table 4. All nominally Significant Results from Single Nucleotide Variant Analysis. The p-value presented is the uncorrected p-value output by SKATBinary_Single.

Supplementary Table 5. All nominally Significant Results from Gene-Level Analysis. The p-value presented is the uncorrected p-value output by SKATBinary.

Supplementary Table 6. All nominally Significant Results from Gene-Set Level Analysis. The P Value presented is the uncorrected p-value output by MAGMA.

Supplementary Table 7. Gene Co-expression Module Associations. The P Value presented is the uncorrected p-value of the hypergeometric test comparing overlap between each gene module and genes associated with TRD at nominal (5%) significance.

Supplementary Table 8. Top Hit Comparison. P values presented from Supplementary Table 4 found in Li et al. 2019. P values presented from Table 6 found in Fabbri et al. 2019.
